# Living with Chronic Kidney Disease and Kidney Transplantation During COVID-19: A Study of Psychological and Behavioral Impacts

**DOI:** 10.3390/healthcare13131488

**Published:** 2025-06-21

**Authors:** Jasmin Jäger, Saskia Reick, Jil Beckord, Peter Weber, Adnan Halilbegovic, Rebekka Bruning, Johanna Reinold, Eva-Maria Skoda, Martin Teufel, Andreas Kribben, Oliver Witzke, Sven Benson, Anja Gäckler, Hana Rohn, Hannah Dinse

**Affiliations:** 1 Department of Infectious Diseases, West German Centre of Infectious Diseases, University Medicine Essen University Hospital Essen, University Duisburg-Essen, 45147 Essen, Germany; saskia.reick@stud.uni-due.de (S.R.); peter.weber@uk-essen.de (P.W.); adnan.halilbegovic@uk-essen.de (A.H.); rebekka.bruning@uk-essen.de (R.B.); oliver.witzke@uk-essen.de (O.W.); hana.rohn@uk-essen.de (H.R.); 2 Clinic for Psychosomatic Medicine and Psychotherapy, University of Duisburg-Essen, LVR-University Hospital, 45147 Essen, Germany; jil.beckord@lvr.de (J.B.); eva.skoda@uk-essen.de (E.-M.S.); martin.teufel@lvr.de (M.T.); hannah.dinse@lvr.de (H.D.); 3 Department of Nephrology, University Medicine Essen University Hospital Essen, University Duisburg-Essen, 45147 Essen, Germany; johanna.reinold@uk-essen.de (J.R.); andreas.kribben@uk-essen.de (A.K.); anja.gaeckler@uk-essen.de (A.G.); 4 Center for Translational Neuro- and Behavioral Sciences (C-TNBS), University of Duisburg-Essen, 45147 Essen, Germany; sven.benson@uk-essen.de; 5 Institute of Medical Psychology and Behavioral Immunobiology, Center for Translational Neuro- and Behavioral Sciences, University Hospital of Essen, 45147 Essen, Germany

**Keywords:** kidney diseases, chronic, COVID-19, anxiety, depression, cross-sectional studies

## Abstract

**Background:** Psychological impacts of the coronavirus disease 2019 (COVID-19) pandemic on individuals with chronic medical conditions remain understudied. Chronic kidney disease (CKD) is one of the chronic conditions associated with an increased risk of severe COVID-19 outcomes. The aim of this study was to define the psychological burden of individuals with CKD during the COVID-19 pandemic. **Methods:** In this cross-sectional study, 219 individuals with CKD were recruited from the Nephrology Outpatient Unit at the University Hospital Essen, Germany, and completed anonymous surveys incorporating validated psychological assessment tools for generalized anxiety (GAD-7) and depressive symptoms (PHQ-2), along with self-generated items addressing COVID-19-specific concerns and behavioral changes. These participants were propensity score matched with controls from the general German population. **Results:** Individuals with CKD exhibited lower levels of generalized anxiety and depressive symptoms compared to the general population. However, they reported significantly greater risk perceptions regarding the likelihood of experiencing symptoms, a severe disease course, and death from COVID-19. COVID-19-related fear and associated behavioral changes were more frequently reported among CKD patients. Adherent and dysfunctional safety behaviors predominated among the CKD cohort. **Conclusions:** This study underscores psychological challenges faced by individuals with CKD during the COVID-19 pandemic. The increased risk perceptions and fears of severe disease and mortality from COVID-19 emphasize the need for mental health interventions aimed at improving coping strategies alongside physical health management in this vulnerable population.

## 1. Introduction

The global coronavirus disease 2019 (COVID-19) pandemic, caused by severe acute respiratory syndrome coronavirus 2 (SARS-CoV-2), continues to present complex health challenges worldwide. While the virus has affected populations broadly, individuals with chronic diseases have faced a markedly elevated risk of severe outcomes from COVID-19 [1]. Chronic conditions such as diabetes, hypertension, cardiovascular disease, and chronic kidney disease (CKD) are particularly associated with higher risks of severe disease progression, complications, and mortality [1]. These risks arise from the combined effects of pre-existing health vulnerabilities, impaired immune responses, and compromised organ function [1].

The pandemic has had substantial implications for individuals with CKD [2,3]. Mounting evidence has demonstrated a heightened susceptibility to COVID-19 infection and increased rates of hospitalization and mortality in this population [4,5,6]. CKD is recognized as both an independent risk factor for severe COVID-19 outcomes and as a condition frequently co-occurring with other chronic illnesses that further amplify COVID-19 risk [3]. Recent studies have highlighted that patients undergoing dialysis face a more than 3.5-fold increased risk of COVID-19-related death compared to those not on dialysis [1]. Kidney transplant recipients (KTRs), a particularly vulnerable subgroup, face critical risks for severe COVID-19 outcomes and mortality due to their impaired immune response from long-term immunosuppressive therapy and high prevalence of comorbidities [7]. CKD patients infected with SARS-CoV-2 experience a notably elevated incidence of cardiovascular complications and cardiovascular-related death [8]. Despite vaccination efforts and emerging therapeutic options, individuals with CKD remain acutely vulnerable to both immediate and long-term adverse health outcomes from COVID-19.

In addition to physical health impacts, the COVID-19 pandemic has had profound and ongoing effects on mental health globally. Numerous studies indicate a significant rise in symptoms of depression, generalized anxiety, and psychological distress during the pandemic in the general population [9,10,11]. Current research suggests long-term effects of the COVID-19 pandemic on mental health at both the individual and societal levels [12,13,14]. These impacts may be amplified among high-risk groups, where the presence of chronic health conditions correlates with increased COVID-19-related anxiety and fear [15,16]. For example, cancer patients reported significantly higher COVID-19-related anxiety [17], and individuals with diabetes demonstrated increased fears regarding symptom severity, disease progression, and mortality associated with COVID-19 [18]. CKD patients already bear an elevated psychological burden for managing their diseases, even in the absence of additional stressors. Their psychological distress is usually elevated due to the complex and progressive nature of kidney disease and the demands of treatment, including frequent medical visits, dietary requirements, and, in advanced cases, reliance on dialysis or immunosuppressive medication following kidney transplantation [19,20,21,22]. In particular, KTRs bear additional layers of psychological burden, including lifelong immunosuppressive therapy, heightened susceptibility to infections, and persistent anxiety about transplant rejection [23]. Elevated levels of anxiety and depression are common among CKD patients and are associated with adverse health outcomes, including increased hospitalization and mortality rates [24,25].

However, the specific psychological burden posed by COVID-19 on individuals with CKD has not been sufficiently studied. This gap highlights the need for research on the pandemic’s mental health impact on this vulnerable population.

The present study aims to quantify levels of generalized anxiety, depressive symptoms, COVID-19-related fear, and safety behaviors among individuals with CKD, especially kidney transplant recipients. The goal is to provide a clearer understanding of the mental health impacts of COVID-19 on CKD patients and to assess the need for targeted interventions to address increased psychological distress in high-risk populations. The findings may aid future mental health support strategies, potentially offering tailored psychological resources to patients with CKD and individuals with similar high-risk chronic conditions in response to pandemic-related psychological needs.

## 2. Materials and Methods

### 2.1. Participants and Study Design

The study used a cross-sectional design based on structured surveys. Between March and September 2021, 232 patients with CKD were recruited from the Nephrology Outpatient Unit at the University Hospital Essen, Germany. Of these, 219 participants were successfully included in the final analysis following propensity score matching with controls from the general German population. A total of 13 CKD patients could not be matched due to the absence of a suitable control with comparable demographic characteristics.

**Figure 1:**
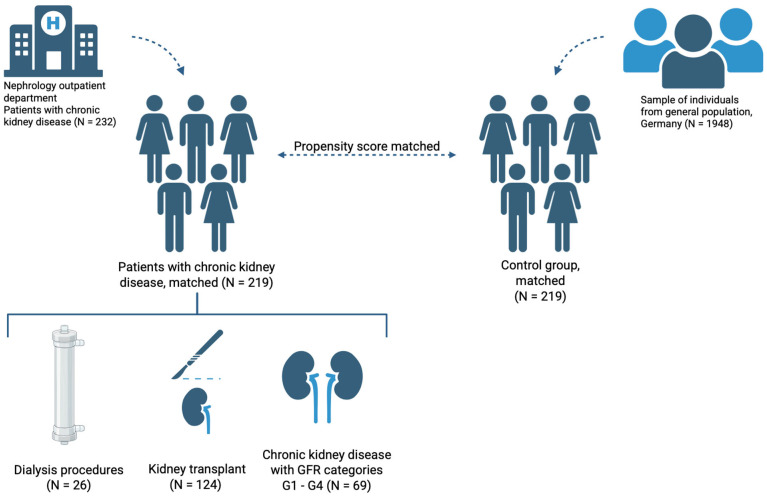
Scheme of the study sample. Dialysis procedures include mainly hemodialysis and peritoneal dialysis. CKD glomerular filtration rate (GFR) categories G1–G4 as defined by Kidney Disease: Improving Global Outcomes (KDIGO) CKD Work Group [26]. Figure created with BioRender.com (accessed on March 2025).

Patients completed questionnaires through structured interviews. The control group was drawn from a large cross-sectional study including 1948 participants within the German general population, as documented in previous research [9]. For the CKD group, inclusion criteria were age 18 years or older and a confirmed diagnosis of chronic kidney disease (any stage, as defined by KDIGO guidelines [26]). Exclusion criteria for both the CKD group and control group were inability to provide informed consent and/or significant cognitive impairment or language barriers that prevented the completion of the structured interview. Controls completed the questionnaires via an online survey between October 2020 and December 2022. An overview of the study sample is depicted in Figure 1. The study was approved by the Ethics Committee of the University Duisburg-Essen (No. 20-9307-BO). Participants completed standardized questionnaires regarding sociodemographic status, previous diseases, and COVID-19-associated effects on psychological burden and health behavior. Missing responses were handled on an item-level basis. Analyses were conducted using all available responses for each item or scale.

### 2.2. Propensity Score Matching

To adjust for potential confounders between CKD patients and the control group, Propensity Score Matching (PSM) was applied to enhance similarity between the groups [27]. PSM helps ensure a balanced distribution of confounding variables across groups, thereby increasing intergroup comparability [27]. This approach reduces the risk that results are influenced by secondary characteristics of the reference group rather than the primary selection criterion.

In this study, PSM was implemented using the nearest-neighbor matching algorithm without replacement in a 1:1 ratio, with propensity scores estimated via logistic regression. Matching was performed without a caliper, and ties among equally distant controls were resolved at random. A sensitivity analysis of the PSM matching is displayed in Figure S1. Controls were matched based on various sociodemographic covariates, including age, sex, marital status, education, and occupational status.

### 2.3. Instruments

Data were collected using standardized questionnaires covering sociodemographic and medical details, validated assessment tools, and custom items addressing COVID-19-specific aspects. Internal consistency and reliability of the survey scales were measured using Cronbach’s alpha [28,29].

Sociodemographic parameters included sex, age, marital status, educational level, and occupational status. Participants with CKD provided additional details on their specific nephrological diagnoses, renal replacement procedures (if applicable), kidney transplantation status or listing, and comorbidities.

Generalized anxiety and depressive symptoms were assessed using the Generalized Anxiety Disorder Scale-7 (GAD-7) and the Patient Health Questionnaire-2 (PHQ-2). Both GAD-7 and PHQ-2 were used in their validated German versions [29,30].

The GAD-7 is a validated tool that screens and quantifies generalized anxiety severity over a two-week period. Participants rate their anxiety on 7 items using a 4-point Likert scale (0–3), resulting in total scores from 0 to 21 (with thresholds at 5 = mild, 10 = moderate, and 15 = severe anxiety).

The GAD-7 demonstrated high reliability and internal consistency in prior research and in this study, with a Cronbach’s α of 0.93 [28,29]. The Patient Health Questionnaire-2 (PHQ-2) assesses depressive mood over the previous two weeks [30]. It includes two items rated on a 4-point Likert scale from 0 (not at all) to 3 (nearly every day), with total scores ranging from 0 to 6. A score of 3 is the cutoff for major depressive symptoms. The PHQ-2 showed good internal consistency (Cronbach’s α of 0.83).

COVID-19-related experience and behaviors such as COVID-19-related fear, personal risk perception, trust in government interventions, and subjective informedness level regarding the disease and its treatment were assessed via self-generated items. Participants rated their subjective level of information about COVID-19, their trust in government measures, and personal risk perceptions on 8 items, using a 7-point Likert scale (1 = strongly disagree, to 7 = strongly agree). This section showed good internal consistency (Cronbach’s α of 0.84).

Adherent Safety Behavior (ASB) and Dysfunctional Safety Behavior (DSB) were measured with 7 items each. ASB included behaviors such as enhanced hygiene and physical distancing, aligning with public health recommendations. DSB included actions such as excessive stockpiling of food or sanitary supplies, which reflect more dysfunctional behaviors [17]. Internal consistency was acceptable, with Cronbach’s α of 0.78 for ASB and 0.83 for DSB.

### 2.4. Statistical Analyses

Propensity score matching was conducted using the R package MatchIt (version 4.5.5, 2023). All other statistical analyses were performed in SPSS Statistics (version 29.0, SPSS Corp., Armonk, NY, USA, 2022). Due to the non-normal distribution of variables, the nonparametric Mann–Whitney U test was applied to compare differences between CKD patients and controls in measures of general anxiety, depressive symptoms, COVID-19-related fear, subjective risk perception, subjective informedness regarding COVID-19, and associated safety behaviors. For multivariate analysis, logistic regression was used to identify factors associated with adherent and dysfunctional safety behaviors. A significance level of α = 0.05 was used to determine statistical significance.

## 3. Results

A total of 219 patients with CKD completed the survey and were matched with 219 controls from the general population based on sociodemographic data. After matching, no statistical differences were observed between the two groups for any baseline variables, with *p* < 0.05 across all variables [31].

Among CKD patients, 46.1% were female, and the majority (53.0%) were between 35 and 64 years old. Of all CKD patients, 71.4% had been diagnosed with the condition for more than five years. KTR made up 56.6% of the CKD cohort. The most commonly reported underlying kidney condition was hypertensive nephropathy (39.3%).

In the control group, 52.5% of the participants were between 35 and 64 years old, and 46.1% were female. Table 1 provides a detailed overview of sociodemographic characteristics for both CKD patients and matched controls, while Table 2 summarizes the medical characteristics of the CKD cohort.

A total of 23.7% of CKD patients scored for mild anxiety, 6.8% for moderate, and 4.7% for severe anxiety symptoms on the GAD-7 scale. In the control group, 29.2% scored mild, 13.2% moderate, and 8.7% severe for anxiety symptoms. Using a cutoff score of 3 on the PHQ-2, 9.1% of all CKD patients and 28.8% of controls reported symptoms indicative of major depression (see Table 3, cut-off value according to [30]).

Corresponding Mann–Whitney-U analyses revealed that differences regarding generalized anxiety and depressive symptoms between CKD patients and the control group are significant, as shown in Table 4. Analysis of GAD-7 and PHQ-2 scores revealed significantly higher levels of generalized anxiety (*p* < 0.01) and depressive symptoms (*p* < 0.001) in the control group compared to the overall CKD cohort.

CKD patients reported significantly higher COVID-19-related fear compared to controls (*p* < 0.001). Regarding the informedness level, CKD patients felt subjectively better informed about COVID-19 than matched controls (*p* < 0.001).

Concerning adherent and dysfunctional safety behaviors, patients with CKD reported higher levels of ASB and DSB compared to healthy controls, as shown in Table 4 (*p* < 0.001).

A significant difference in the subjective risk perception of contracting COVID-19 was not found between CKD patients and controls (see Table 5). In contrast, CKD patients reported a significantly higher perceived individual risk of experiencing COVID-19-associated symptoms (*p* < 0.001), suffering a severe course (*p* < 0.001), or death from COVID-19 (*p* < 0.001), as shown in Table 5.

A multivariate logistic regression analysis revealed that both adherent and dysfunctional safety behaviors are strongly predicted by COVID-19-related fear, as shown in Table 6.

COVID-19-related fear had the strongest positive association with adherent safety behavior (*p* < 0.001), followed by subjective informedness (*p* < 0.001) and subjective risk perception of contracting the virus (*p* < 0.01). COVID-19-related fear (*p* < 0.001) and subjective risk perception (*p* < 0.05) are also significant predictors of dysfunctional safety behavior, with rather small effect sizes. The subjective level of informedness about COVID-19 did not predict DSB.

## 4. Discussion

To date, essential research on the psychological burden of individuals with chronic kidney disease due to COVID-19 remains lacking. This study is the first to examine the psychological and behavioral impact of the COVID-19 pandemic on patients with CKD, compared to matched controls from the general population in Germany. Unexpectedly, individuals with CKD did not exhibit heightened symptoms of generalized anxiety or depression. Both CKD patients and controls revealed similar individual risk perceptions of an infection with SARS-CoV-2. However, CKD patients reported significantly higher risk perceptions of the occurrence of symptoms, a severe course, and death from COVID-19. COVID-19-related fear as well as associated behavioral changes were also more frequently reported among CKD patients.

Chronic kidney disease was identified early as one of the chronic conditions classified as high risk factors for severe COVID-19 outcomes, contributing to high rates of hospitalization and mortality [32,33,34]. Despite a heightened psychological burden due to their underlying nephrological condition, we hypothesized that CKD patients were particularly affected mentally by the COVID-19 pandemic.

The present work did not yield an increased level of generalized anxiety or depressive symptoms in CKD patients compared to propensity-matched controls from the general population.

During the pandemic, the overall prevalence of generalized anxiety disorder and depression increased among the general population in many countries [35]. In Germany, prevalence rates of generalized anxiety (depressive symptoms) were observed between 22 and 24% (33 and 34%) during the second lockdown period from December 2020 to March 2021 [36].

Consistent with these findings, the results from this study reflect a sharp increase in mental health disorders among both individuals with CKD and controls from the general population in Germany during the COVID-19 pandemic. This result highlights an additional psychological burden due to COVID-19 that is independent of pre-existing chronic conditions, even though the present study was conducted shortly after Germany’s second lockdown period when public restrictions were being eased, and vaccinations were well-established and prioritized for vulnerable groups such as CKD patients.

CKD patients scored relatively lower than controls on both assessment instruments, GAD-7 and PHQ-2, suggesting that CKD patients might experience fewer symptoms of generalized anxiety and depression than the general population during COVID-19. This finding is somewhat surprising, given the significantly elevated risk of severe COVID-19 outcomes in CKD patients, as well as the potentially higher psychological burden associated with their underlying chronic kidney condition [21,22]. The comparatively lower scores in anxiety and depressive symptoms within the CKD cohort could be attributed to the more effective and comprehensive provision of psychological support services available to this group. CKD patients, particularly kidney transplant recipients, attend regular medical check-ups with their nephrologist at least four times a year. Due to their more frequent contact with healthcare facilities, we assume that these individuals may have better access to a wider range of medical services, including prioritized access to COVID-19 vaccines, and quicker provision of psychological support [37].

The elevated COVID-19-related fear among CKD patients appears to stem from their heightened risk perception. Although the perceived likelihood of infection is rated similarly in both CKD patients and controls, CKD patients report a significantly greater perceived risk of developing symptoms, experiencing a severe course of disease, and death from COVID-19 compared to the general population. This finding is plausible, as chronic conditions like CKD are known to predispose individuals to severe COVID-19 complications and increased mortality [1,7]. The realistic assessment of personal risk among CKD patients, along with efforts by care providers to increase awareness of these risks, likely contributes to their heightened emotional response, manifesting as increased fear related to COVID-19.

CKD patients in this study reported higher levels of subjective informedness about COVID-19 compared to controls, suggesting that CKD patients feel more adequately informed about the disease, its progression, potential complications, and effective protective measures. Research indicates that a higher subjective level of informedness is often associated with reduced emotional distress and psychological burden, alongside increased self-efficacy, emphasizing the role of information provision for vulnerable groups [38,39]. The findings of the present study seem to underline these results, since CKD patients had lower levels of anxiety and depressive symptoms compared to controls from the general population. The observations suggest that the comprehensive care CKD patients typically receive through close contact with healthcare providers may contribute to both their greater subjective informedness and realistic risk assessment. We, therefore, suggest that better-informed CKD patients may experience a greater sense of control and preparedness regarding COVID-19 risks, which can help alleviate some of the associated psychological stress. Future care models for vulnerable populations may benefit from incorporating scalable mental health support and telehealth solutions to ensure continuity of care and address psychosocial needs during crises such as pandemics. The addition of qualitative data, such as patient narratives, could complement the findings of this work and offer deeper insights into the lived experiences and psychosocial challenges of individuals with CKD during the pandemic. Future mixed-methods studies could explore these aspects more comprehensively.

Beyond psychological burden, several studies have shown that the COVID-19 pandemic has led to notable behavioral changes among vulnerable populations, particularly those with chronic somatic conditions [17,18]. Behavioral responses, including adherent safety behaviors and maladaptive, dysfunctional safety behaviors, often arise from emotional reactions and cognitive evaluations of public health threats.

In this study, CKD patients displayed significantly higher levels of ASB and DSB than controls from the general population. Patients with CKD reported increased ASB, such as frequent handwashing, use of disinfectants, and avoidance of public spaces—behaviors that align with public health recommendations aimed at minimizing infection risk. However, CKD patients also exhibited higher levels of DSB, such as stockpiling essential items, compared to healthy controls. This pattern suggests a dual reaction, where protective behaviors coexist with potentially excessive or maladaptive responses to perceived threats.

COVID-19-related fear and subjective risk perception predicted both adherent and dysfunctional safety behaviors in this study. Beyond the fear of COVID-19, the heightened subjective risk perception among CKD patients is presumed to primarily drive engagement in ASB and DSB, leading to overcompensation through both protective and excessive actions. This aligns with previous studies indicating that individuals who perceive themselves as more vulnerable or at greater risk of severe outcomes from COVID-19 are more likely to engage in frequent safety practices [40,41].

Although the subjective level of informedness significantly predicted ASB, it had no such effect on DSB, highlighting that fear can be a powerful emotional response that bypasses rational, informed decision-making processes, leading to irrational and maladaptive behaviors such as panic buying of staple foods [42].

These results support previous research findings that chronic diseases contribute to more intense and varied behavioral responses to health threats [17,18]. In the case of CKD patients, DSB may reflect an amplified attempt to prepare for potential health crises, possibly stemming from heightened anxiety associated with their chronic condition and its risks.

Data collection took place during a period when the wild-type and Alpha variants of SARS-CoV-2 were predominant in Germany. As later variants (e.g., Delta, Omicron) differed in transmissibility and disease severity, psychological responses may have evolved in parallel with these changes. Future studies should consider the influence of specific viral variants on mental health outcomes in vulnerable populations such as individuals with CKD.

Overall, our findings underscore the complex interplay between fear, risk perception, and behavior among vulnerable groups. For patients with chronic conditions like CKD, targeted interventions such as tailored health information and psychological support for emotional regulation could be beneficial. Providing accurate information on COVID-19 risks and emphasizing adherent rather than excessive or dysfunctional behaviors might reduce anxiety-driven actions while still promoting effective safety practices. Additionally, mental health support and counseling aimed at mitigating excessive fear could help CKD patients navigate the pandemic more confidently, balancing realistic precautions with psychological well-being.

### Limitations

This study faces certain limitations. The cross-sectional design of the study precludes any conclusions about causality, making it difficult to determine whether the observed psychological effects are a direct result of the pandemic or if they existed prior to it. To enhance future research, it would be beneficial to utilize longitudinal designs that can better assess causal relationships.

Additionally, the reliance on self-reported data means that there is no objective verification, which limits the ability to accurately confirm diagnoses, symptoms, or behaviors.

A key methodological limitation concerns the differing modes of data collection between groups. CKD patients participated in structured, face-to-face interviews, where interviewers could clarify questions and prompt more complete responses. In contrast, controls completed online surveys independently, which may increase the likelihood of misunderstanding questions or skipping items. This introduces the risk of systematic differences in how data were generated across groups, potentially affecting comparability. At the same time, face-to-face interviews may introduce social desirability bias, as participants might modify their responses due to the presence of an interviewer. These differences in administration could have influenced the reported levels of anxiety, fear, or behavioral responses.

Vaccination status was not assessed in this study. Given the ongoing vaccination campaign during the data collection period, individual differences in vaccine access and uptake may have influenced participants’ risk perceptions and psychological responses.

Furthermore, at the time this study was conducted, a brief, validated instrument to assess COVID-19-related fear, such as the Fear of COVID-19 Scale (FCV-19S), had not yet been established [43]. Instead, a self-generated single-item measure was utilized, which may raise concerns about its validity and reliability in capturing the nuances of fear associated with COVID-19.

## 5. Conclusions

This study highlights the psychological and behavioral responses of individuals with chronic kidney disease during the COVID-19 pandemic. Despite the known elevated risk of severe COVID-19 outcomes, the findings indicate that CKD patients do not exhibit higher levels of generalized anxiety or depressive symptoms than matched controls from the general population, suggesting that comprehensive healthcare support systems available to CKD patients play a significant role in their mental health resilience during the pandemic.

CKD patients demonstrated heightened COVID-19-related fear and a realistic assessment of their personal health risks, which corresponded to increased adherence to safety behaviors. However, they also engaged in dysfunctional safety behaviors, illustrating the complex interplay between risk perception, emotional response, and behavior in the face of public health threats.

A higher level of subjective informedness among CKD patients indicates that access to information and healthcare support may enhance their sense of control and preparedness regarding COVID-19 risks, emphasizing the importance of information provision in reducing psychological distress and promoting self-efficacy among vulnerable populations.

In light of these findings, it is essential to continue prioritizing mental health support for CKD patients, especially in the context of the long-term impacts of the pandemic on this population. Providing CKD patients with tailored information, emotional support, and integrated care strategies will enable healthcare providers to mitigate psychological distress, promote resilience, and improve overall health outcomes in an increasingly uncertain environment.

## Figures and Tables

**Table 1:** Sociodemographic data of CKD patients and controls. *p*-values calculated using Chi-square test.

	CKD Patients		Matched Controls from General Population		
Characteristics	N = 219	%	N = 219	%	*p*
**Sex**					1.000
Female	101	46.1	101	46.1	
Male	118	53.9	118	53.9	
**Age**					0.777
18–34	52	23.7	55	25.1	
35–64	116	53.0	115	52.5	
65–84	50	22.8	49	22.4	
85+	1	0.5	0	0	
**Marital status**					0.216
Single	53	24.2	67	30.6	
Married	114	52.1	109	49.8	
In a relationship	22	10.0	23	10.5	
Divorced/separated	20	9.1	17	7.8	
Widowed	10	4.6	3	1.3	
**Educational level**					0.019
University education	38	17.4	40	18.3	
Higher education entrance qualification	49	22.4	73	33.3	
Intermediate secondary education	59	26.9	61	27.9	
Lower secondary education	63	28.7	34	15.5	
No qualification	5	2.3	5	2.3	
Other	5	2.3	6	2.7	
**Employment**					0.767
Full employment	83	37.9	80	36.5	
Not employed	136	62.1	139	63.5	

**Table 2:** CKD-associated characteristics, comorbidities, and mental disorders of CKD patients. CKD glomerular filtration rate (GFR) categories G1–G4 as defined by Kidney Disease: Improving Global Outcomes (KDIGO) CKD Work Group [26].

CKD Patient Characteristics	N = 219	%
**Renal disease**		
Arterial hypertension	86	39.3
Glomerulonephritis	31	14.2
Cystic kidney disease	30	13.7
Diabetes mellitus	24	11.0
Systematic disease	20	9.1
Renal vascular diseases	7	3.2
Hemolytic uremic syndrome (HUS)	7	3.2
Others	22	10.0
More than one renal disease	8	3.7
**Dialysis**	26	11.9
Hemodialysis	18	8.2
Peritoneal dialysis	8	3.7
**Kidney transplantation**	124	56.6
**CKD GFR categories G1–G4** (Without dialysis or kidney transplantation)	69	31.5
**Comorbidities**		
Cardiovascular	30	13.7
Diabetes	26	11.9
Chronic respiratory diseases	20	9.1
Arterial hypertension	113	51.6
Other	19	8.7
Not specified/unknown	25	11.4
More than 2 comorbidities	29	13.2
More than 3 comorbidities	6	2.7

**Table 3:** Prevalence of generalized anxiety (GAD-7) and depressive symptoms (PHQ-2) in CKD patients and controls. GAD-7 sum scores of ≥5, ≥10, and ≥15 indicate mild, moderate, and severe generalized anxiety symptoms, respectively. PHQ-2 sum scores of ≥3 indicate major depression symptoms. NA: No answer provided.

	CKD All (N = 219)	Controls (N = 219)
**GAD-7**		
No anxiety (<5)	124 (56.6%)	107 (48.9%)
Mild anxiety (≥5 < 10)	52 (23.7%)	64 (29.2%)
Moderate anxiety (≥10 < 15)	15 (6.8%)	29 (13.2%)
Severe anxiety (≥15)	10 (4.7%)	19 (8.7%)
NA	18 (8.2%)	0
**PHQ-2**		
No depression (<3)	133 (60.7%)	156 (71.2%)
Major depression (≥3)	20 (9.1%)	63 (28.8%)
NA	66 (30.2%)	0

**Table 4:** Comparison of CKD patients and healthy controls regarding different psychological assessment instruments, including generalized anxiety (GAD-7), depressive symptoms (PHQ-2), adherent safety behavior (ASB), and dysfunctional safety behavior (DSB) using Mann–Whitney-U analysis (M as mean parameter values and SD as standard deviation).

	CKD Patients (N = 219)	Controls (N = 219)	
	M (±SD)	M (±SD)	*p*
GAD-7	4.26 (4.65)	5.68 (5.34)	<0.01
PHQ-2	1.14 (1.43)	1.87 (1.89)	<0.001
COVID-19-related fear	3.65 (1.89)	2.77 (2.14)	<0.001
Subjective level of Information	5.01 (1.11)	4.11 (1.56)	<0.001
ASB	4.07 (1.59)	3.05 (1.92)	<0.001
DSB	2.02 (1.45)	1.44 (1.21)	<0.001

**Table 5:** Comparison of CKD patients and healthy controls regarding subjective risk perception about COVID-19 in CKD using Mann–Whitney-U analysis with M as mean parameter values and SD as standard deviation.

	CKD Patients (N = 219)	Controls (N = 219)	
Subjective Risk Perception	M (±SD)	M (±SD)	*p*
COVID-19 infection	26.63 (22.82)	25.47 (25.78)	0.195
Symptoms of COVID-19	63.37 (31.05)	44.75 (33.76)	<0.001
Severe course of COVID-19	51.82 (31.76)	26.74 (29.19)	<0.001
Death from COVID-19	34.69 (31.92)	17.13 (24.98)	<0.001

**Table 6:** Linear regression analysis of variables predicting adherent safety behavior (ASB) and dysfunctional safety behavior (DSB) from the total sample (CKD patients and controls) with B as unstandardized regression coefficient and ß as standardized coefficient (strength of the association in standard deviation units).

	Variables	B	ß	*p*
**ASB**	COVID-19-related fear	0.027	0.344	<0.001
	Subjective level of informedness	0.105	0.307	<0.001
	Subjective risk perception (infection)	0.002	0.125	<0.01
**DSB**	COVID-19-related fear	0.009	0.181	<0.001
	Subjective level of informedness	−0.007	−0.029	0.558
	Subjective risk perception (infection)	0.002	0.116	<0.05

## Data Availability

The data presented in this study are available on request from the corresponding author. The data are not publicly available due to privacy or ethical restrictions.

## Supplementary Materials

The following supporting information can be downloaded at: https://www.mdpi.com/article/10.3390/healthcare13131488/s1. Figure S1. Standardized mean differences (SMD) for covariates included in the propensity score model before (open circles) and after (filled circles) matching between individuals with CKD and controls from the general population. Vertical dashed lines indicate thresholds for acceptable balance (|SMD| < 0.05 and < 0.1). Post-matching SMDs fall well below the 0.1 threshold for all covariates, indicating successful covariate balance and robust matching.
