# Supplementary material for: Living with Chronic Kidney Disease and Kidney Transplantation During COVID-19: A Study of Psychological and Behavioral Impacts
Source: Healthcare (Basel). 2025 Jun 21;13(13):1488. doi: 10.3390/healthcare13131488 (PMC12250486; doi:10.3390/healthcare13131488)
Supplement: Supplementary file 1 [file healthcare-13-01488-s001.zip › healthcare-3640367-supplementary.pdf]

healthcare

MDPI

Supplementary material

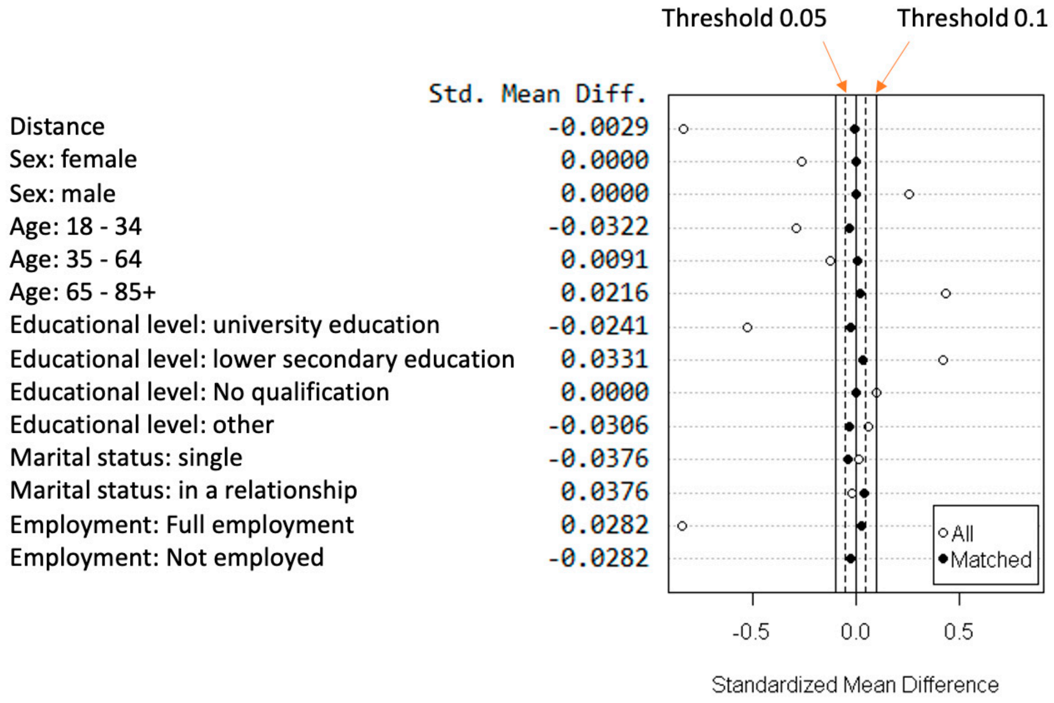

Figure S1. Standardized mean differences (SMD) for covariates included in the propensity score model before (open circles) and after (filled circles) matching between individuals with CKD and controls from the general population. Vertical dashed lines indicate thresholds for acceptable balance (|SMD| < 0.05 and < 0.1). Post-matching SMDs fall well below the 0.1 threshold for all covariates, indicating successful covariate balance and robust matching.
